# Supplementary material for: Nur77 protects the bladder urothelium from intracellular bacterial infection
Source: Nat Commun. 2024 Sep 27;15:8308. doi: 10.1038/s41467-024-52454-8 (PMC11436794; doi:10.1038/s41467-024-52454-8)
Supplement: Supplementary file 1 — Supplementary Information [file 41467_2024_52454_MOESM1_ESM.pdf]

SUPPLEMENTARY FIGURES

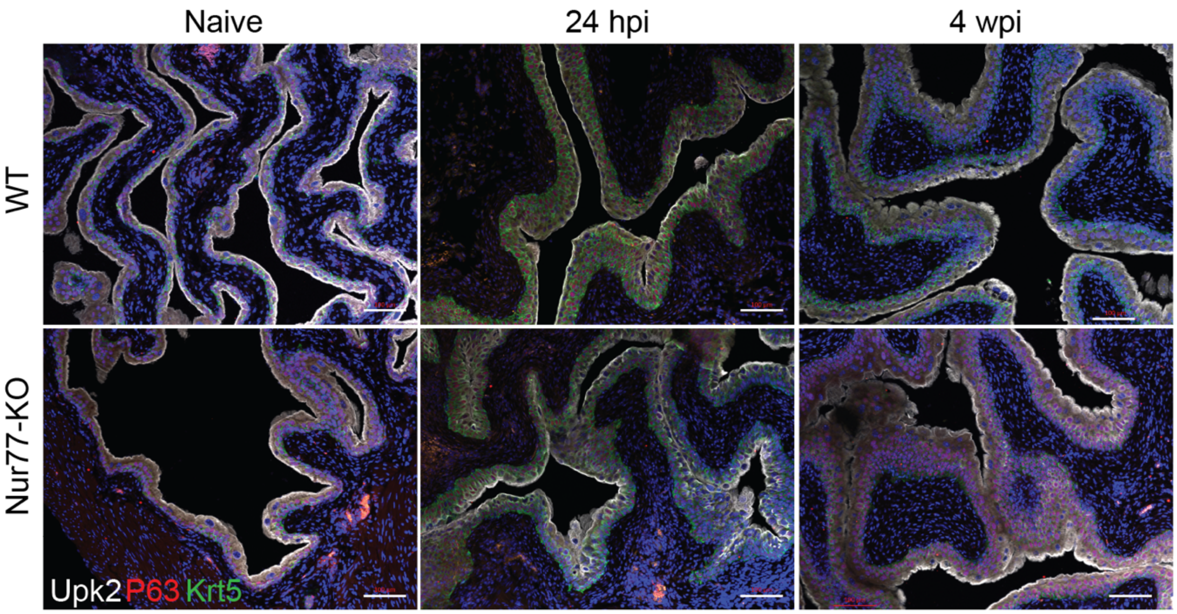

Supplementary Figure 1. WT and Nur77-KO mice have similar urothelial architecture prior to, during, and after UTI. Immunofluorescence microscopy of bladder sections stained with primary antibodies against uroplakin 2 (Upk2, white), p63 (red) and keratin 5 (Krt5, green) and nuclei stained with DAPI (blue). Scale bars = 100 μm.

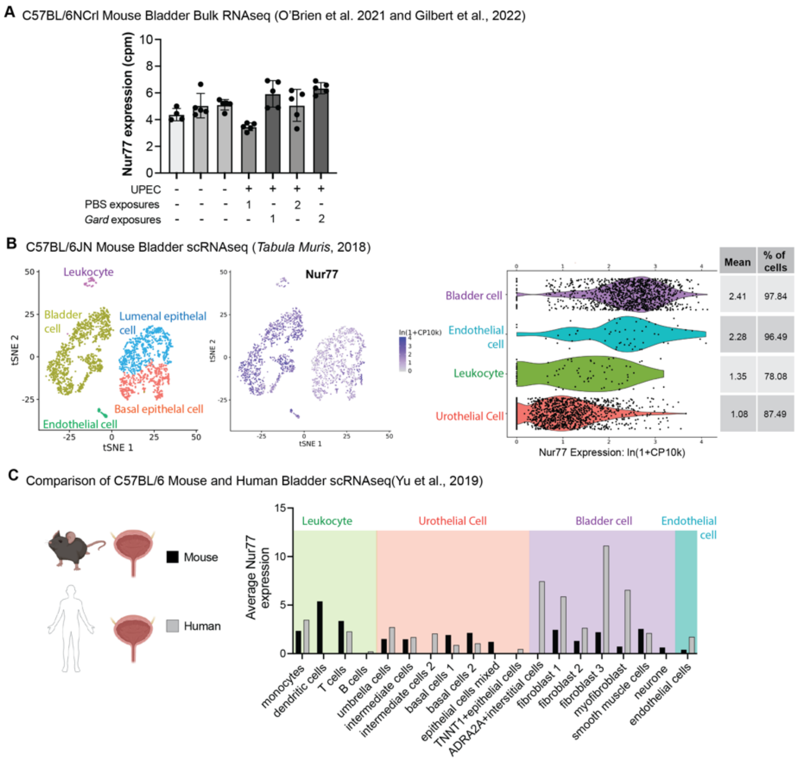

Supplementary Figure 2. Nur77 is expressed by multiple cell types in the mouse and human bladder. (A) Nur77 expression levels in bladders from mice infected with UPEC for 4 weeks or given urinary tract exposures to either PBS or *Gardnerella*, as indicated. n = 5 mice per group. (B) tSNE plot representation of 2,500 urinary bladder cells analyzed by microfluidic droplet-based 3'-end counting from *Tabula Muris*, with cell type annotation (left) and Nur77 expression (right). Right panel shows Nur77 expression levels and frequencies in the four bladder cell clusters from *Tabula Muris*. Images in A and B obtained from https://tabula-muris.ds.czbiohub.org. (C) Nur77 expression in 12,884 mouse and 12,423 human urinary bladder cells analyzed by high-throughput, droplet-based scRNAseq, segregated by distinct cell clusters as annotated by Yu et al. Supplementary Figure 2/panel C created with BioRender.com released under a Creative Commons Attribution-NonCommercial-NoDerivs 4.0 International license. Background colors on the bar graph indicate broader cell type categories to facilitate comparison of the two datasets.

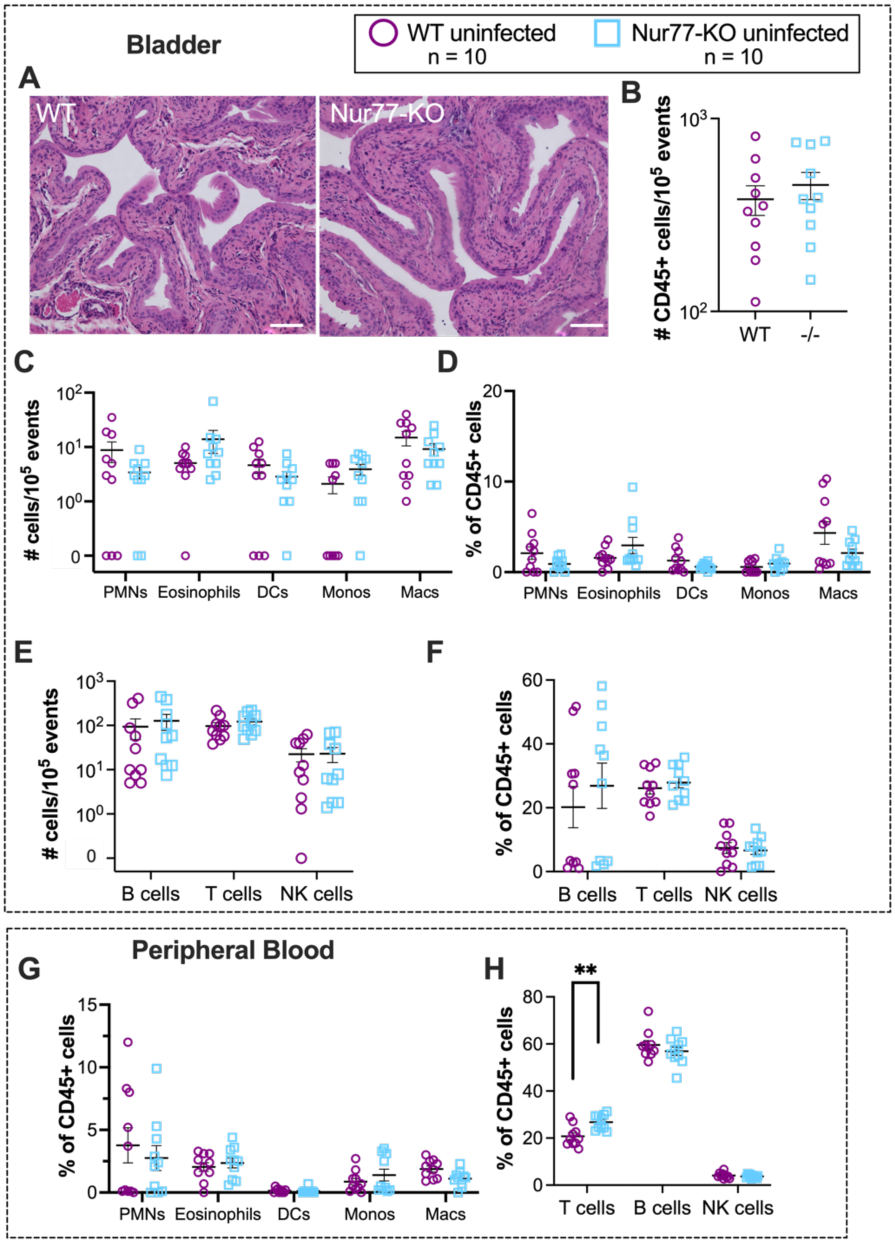

Supplementary Figure 3. Immune cell populations in the bladder are not altered in uninfected Nur77-KO mice. (A) H&E staining of formalin-fixed paraffin bladder tissue sections from naïve mice. Scale bar = 100 µm. (B) Numbers of CD45+ cells in naïve bladders. (C) Numbers of myeloid cell types in naïve bladders. (D) Relative abundance of myeloid cell types in naïve bladders. (E) Numbers of lymphoid cell types in naïve bladders. (F) Relative abundance of lymphoid cell types in naïve bladders. (G) Relative abundance of myeloid and (H) lymphoid cell types in peripheral blood. n = 10 mice per group. Data are combined from 2 independent experiments. ** P = 0.002273 Mann-Whitney with Holm-Šídák method.

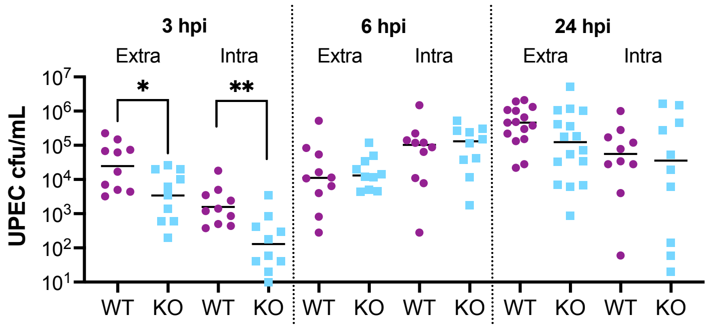

Supplementary Figure 4. Acute UPEC infection gentamicin protection assays. Bladders from UPEC infected wild time (WT) and Nur77-KO (KO) mice were harvested 3, 6, and 24 hpi. Bladders were quadrisected and washed with PBS to enumerate extracellular (Extra) UPEC in the bladder lumen. Following incubation in gentamicin to kill remaining adherent extracellular bacteria, bladders were homogenized to enumerate intracellular (Intra) UPEC in the bladder tissue. **** $P < 0.0001$ Kruskal-Wallis; ** $P = 0.0023$, * $P = 0.0411$ by two-tailed Mann-Whitney test. n = 10 mice per group per time point, combined from two independent experiments.

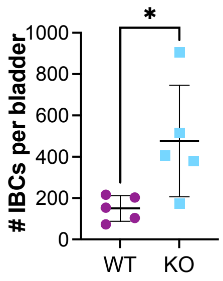

Supplementary Figure 5. UPEC produce more intracellular bacterial communities (IBCs) in the bladders of Nur77-KO compared to WT mice. Data from an independent experiment performed as in Fig 3B. Bladders collected 6 hpi and stained with X-gal to visualize IBCs, which were enumerated. * $P = 0.0302$ by two-tailed Unpaired t test. n = 5 mice per group.

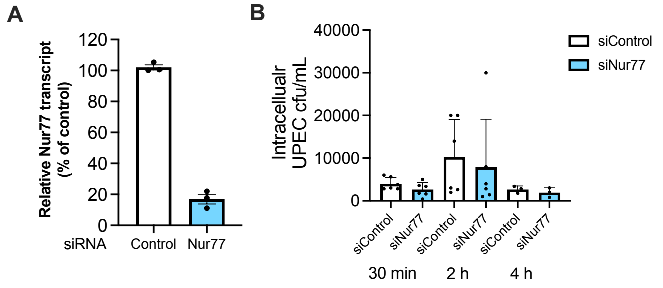

Supplementary Figure 6. Effect of siRNA of Nur77 on UPEC intracellular infection of 5637 urothelial cells. (A) qPCR of Nur77 in 5637 cells transfected with control siRNA or Nur77 siRNA. (B) Time course of intracellular UPEC titers in siRNA cells. n = 3 biological replicates

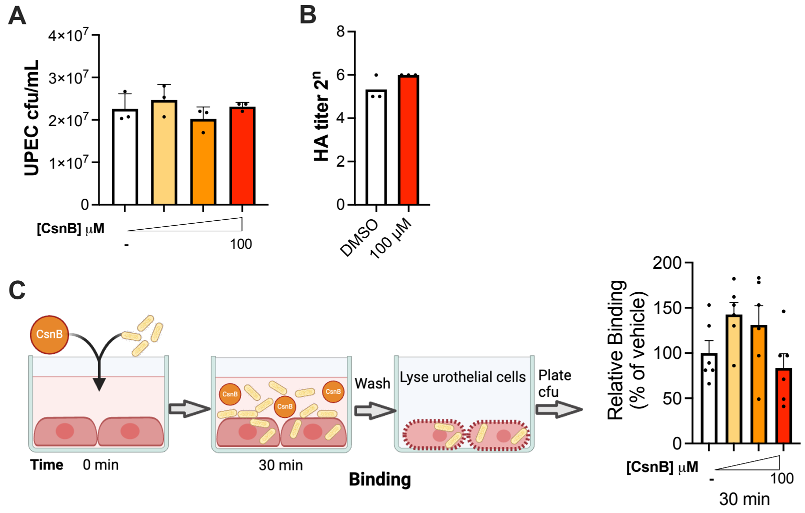

Supplementary Figure 7. Effect of CsnB on UPEC cell viability and hemagglutination. (A) UPEC cfu following 30 min incubation with the indicated concentrations of CsnB. N = 3 biological replicates per condition. (B) Guinea pig red blood cell hemagglutination (HA) titers for UPEC in the presence of DMSO or 100 μM CsnB. n = 3 biological replicates per condition. (C) Relative binding of UPEC to 5637 cells 30 min after incubation. n = 6 biological replicates per condition, combined from two independent experiments. Supplementary Figure 7/panel C schematic created with BioRender.com released under a Creative Commons Attribution-NonCommercial-NoDerivs 4.0 International license.

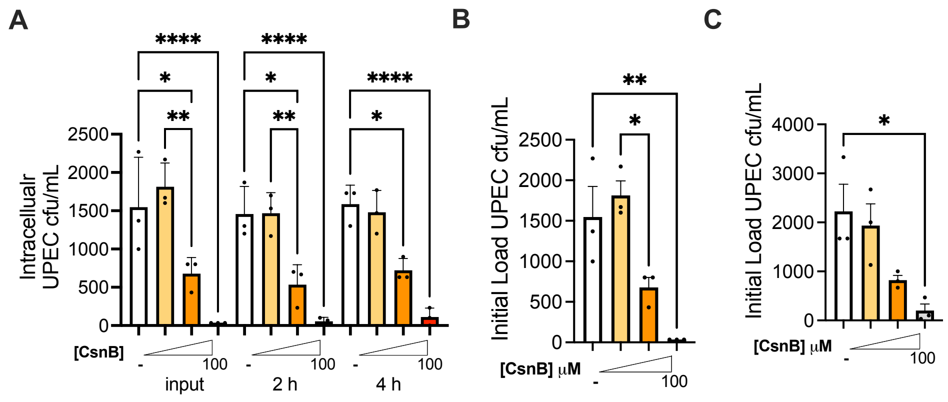

Supplementary Figure 8. Cytosporone B inhibits UPEC intracellular infection of 5637 urothelial cell line *in vitro*. (A) Independent experiment of time course of intracellular UPEC titers in cells treated concurrently with DMSO vehicle control (-) or increasing doses of CsnB. Independent experimental replicate of that shown in Fig 6C. **** $P < 0.0001$, ** $P = 0.0097$ (2 hpi 1 vs 10 μM) ** $P = 0.0011$ (input vs 10 μM), * $P = 0.0191$ (input DMSO vs 10 μM), * $P = 0.0108$ (2 hpi DMSO vs 10 μM), * $P = 0.0200$ (4 hpi DMSO vs 10 μM) by one-way ANOVA with Šídák's multiple comparisons test. (B-C) Independent experiments measuring the initial load of intracellular UPEC titers 30 min post inoculation with concurrent CsnB treatment. ** $P = 0.0058$, * P = 0. 0303 (B) * $P = 0.0219$ (C) by one-way ANOVA with Šídák's multiple comparisons test. All data are from three biological replicates for each condition. n = 3 biological replicates per condition and time point.

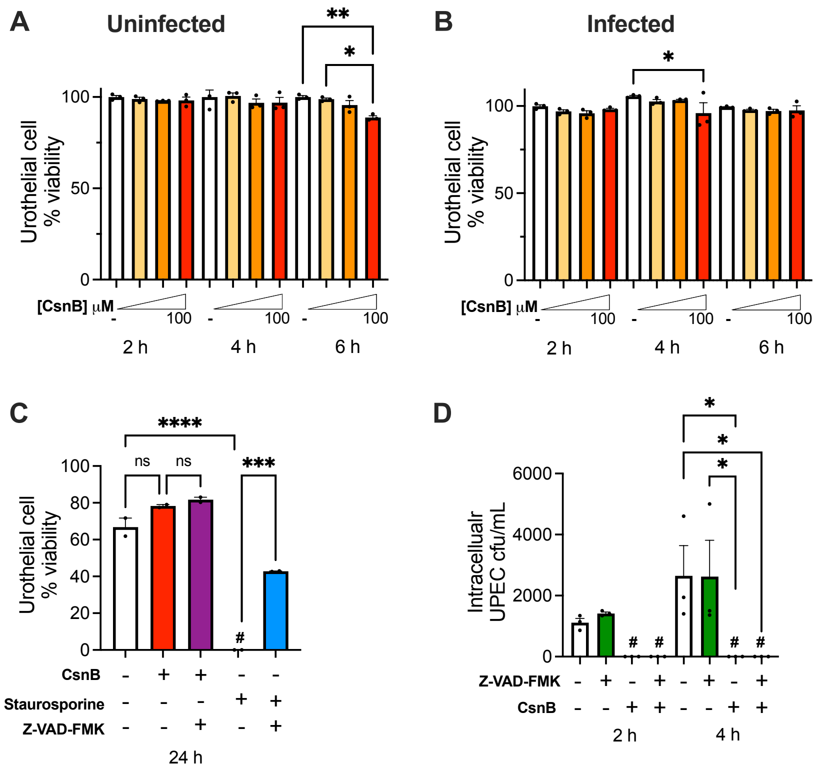

Supplementary Figure 9. Cytosporone B inhibition of UPEC intracellular infection of 5637 urothelial cell line is not due to induction of cell death. (A) Time course of viability of 5637 cells treated with increasing doses of CsnB relative to DMSO vehicle control (-). ** P = 0.0054, * P = 0.0167 by one-way ANOVA with Šídák's multiple comparisons test. n = 3 biological replicates per condition and time point. (B) Time course of viability of 5637 cells concurrently infected with UPEC and treated with increasing doses of CsnB relative to DMSO vehicle control (-). * P = 0.0387 by one-way ANOVA with Šídák's multiple comparisons test. n = 3 biological replicates per condition and time point. (C) Viability of 5637 cells treated with CsnB, staurosporine (induces apoptosis) or Z-VAD-FMK (pan-caspase inhibitor) relative to DMSO vehicle control (-).**** P < 0.0001, *** P = 0.0002 by one-way ANOVA with Šídák's multiple comparisons test. n = 2 biological replicated per condition. (D) Intracellular UPEC cfu in 5637 cells treated with CsnB and/or Z-VAD-FMK. * P = 0.0357 (untreated vs. treated) * P = 0.0383 (Z-VAD-FMK vs. CsnB) by one-way ANOVA with Šídák's multiple comparisons test. n = 3 biological replicates per condition and time point.

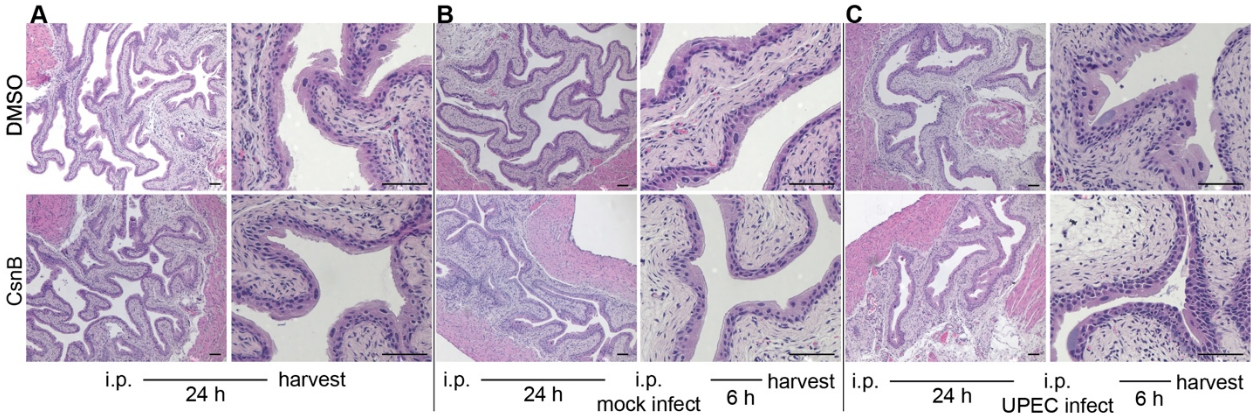

Supplementary Figure 10. Cytosporone B treatment does not cause overt damage or inflammation in bladder tissue. H&E-stained formalin-fixed, paraffin embedded bladder sections from mice treated i.p. with vehicle (DMSO) or CsnB in the same manner as for the UTI experiments in Fig 7. **(A)** Bladders harvested 24 h after a single i.p. injection. This represents the time point at which UPEC would be introduced into the bladder. **(B)** Bladders harvested 6 h after a second i.p. injection and transurethral inoculation of PBS. This examines the effect of two CsnB injections independent from UPEC infection. **(C)** Bladders harvested 6 h after a second i.p. injection and transurethral inoculation of UPEC. This represents the time point at which IBCs are enumerated.

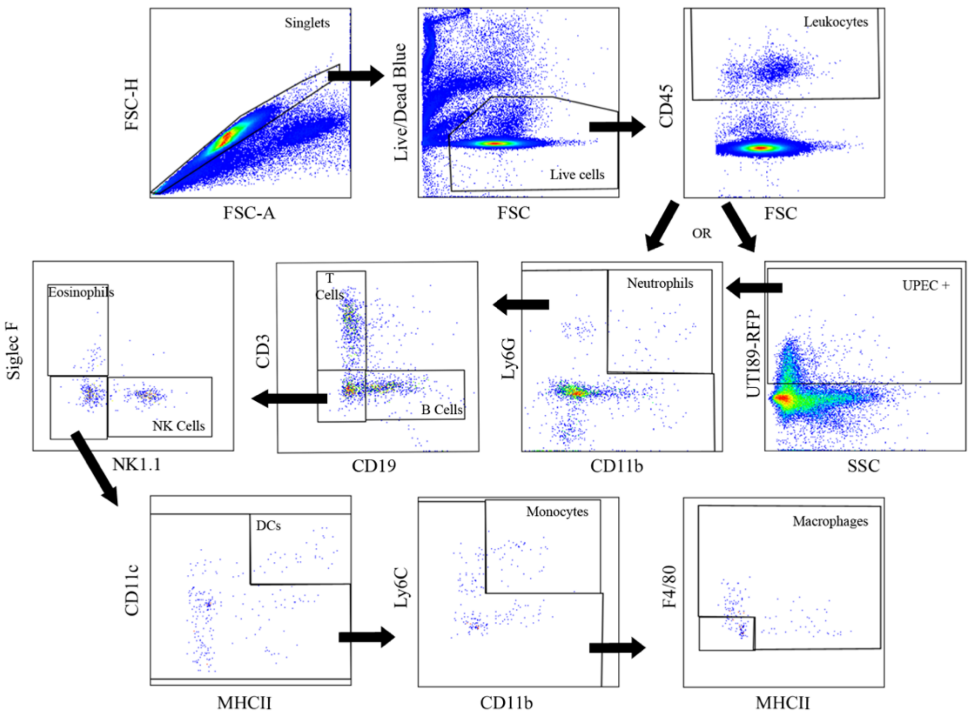

Supplementary Figure 11. Flow cytometry gating strategy.

SUPPLEMENTARY TABLES

Supplementary Table 1. Antibodies used for flow cytometry

Antibody	Manufacturer	Product number
CD45 BV510	Becton Dickinson	563891
I-A/I-E (MHCII) BUV395	Becton Dickinson	743876
CD19 BUV661	Becton Dickinson	612971
CD3 BUV805	Becton Dickinson	741982
CD11b BV570	BioLegend	101233
F4/80 BB700	Becton Dickinson	746070
NK-1.1 PE/Dazzle 594	BioLegend	108748
Ly-6C PE-Cy5.5	Novus Biologicals	N100-65413PECY55
Siglec-F BV421	BioLegend	155509
Ly6-G Alexa Fluor 700	BioLegend	127621
CD11c BV785	BioLegend	117336
