# Supplementary material for: Nur77 protects the bladder urothelium from intracellular bacterial infection
Source: Nat Commun. 2024 Sep 27;15:8308. doi: 10.1038/s41467-024-52454-8 (PMC11436794; doi:10.1038/s41467-024-52454-8)
Supplement: Supplementary file 3 — Reporting Summary [file 41467_2024_52454_MOESM3_ESM.pdf]

nature portfolio

Corresponding author(s): *Double-anonymous peer review submissions: write DAPR and your manuscript number here instead of author names.*

Last updated by author(s): *YYYY-MM-DD*

Reporting Summary

Nature Portfolio wishes to improve the reproducibility of the work that we publish. This form provides structure for consistency and transparency in reporting. For further information on Nature Portfolio policies, see our Editorial Policies and the Editorial Policy Checklist.

Statistics

For all statistical analyses, confirm that the following items are present in the figure legend, table legend, main text, or Methods section.

n/a	Confirmed	
☐	☒	The exact sample size (n) for each experimental group/condition, given as a discrete number and unit of measurement
☒	☐	A statement on whether measurements were taken from distinct samples or whether the same sample was measured repeatedly
☐	☒	The statistical test(s) used AND whether they are one- or two-sided<br>*Only common tests should be described solely by name; describe more complex techniques in the Methods section.*
☒	☐	A description of all covariates tested
☐	☒	A description of any assumptions or corrections, such as tests of normality and adjustment for multiple comparisons
☐	☒	A full description of the statistical parameters including central tendency (e.g. means) or other basic estimates (e.g. regression coefficient) AND variation (e.g. standard deviation) or associated estimates of uncertainty (e.g. confidence intervals)
☒	☐	For null hypothesis testing, the test statistic (e.g. F, t, r) with confidence intervals, effect sizes, degrees of freedom and P value noted<br>*Give P values as exact values whenever suitable.*
☒	☐	For Bayesian analysis, information on the choice of priors and Markov chain Monte Carlo settings
☒	☐	For hierarchical and complex designs, identification of the appropriate level for tests and full reporting of outcomes
☒	☐	Estimates of effect sizes (e.g. Cohen's d, Pearson's r), indicating how they were calculated

Our web collection on statistics for biologists contains articles on many of the points above.

Software and code

Policy information about availability of computer code

Data collection	*Provide a description of all commercial, open source and custom code used to collect the data in this study, specifying the version used OR state that no software was used.*
Data analysis	*Provide a description of all commercial, open source and custom code used to analyse the data in this study, specifying the version used OR state that no software was used.*

For manuscripts utilizing custom algorithms or software that are central to the research but not yet described in published literature, software must be made available to editors and reviewers. We strongly encourage code deposition in a community repository (e.g. GitHub). See the Nature Portfolio guidelines for submitting code & software for further information.

Data

Policy information about availability of data

All manuscripts must include a data availability statement. This statement should provide the following information, where applicable:

- Accession codes, unique identifiers, or web links for publicly available datasets
- A description of any restrictions on data availability
- For clinical datasets or third party data, please ensure that the statement adheres to our policy

Single cell RNAseq data shown in Fig. S2 are available in the previously published studies https://tabula-muris.ds.czbiohub.org and (38). All data generated in this study are provided in the Source Data file.

Research involving human participants, their data, or biological material

Policy information about studies with human participants or human data. See also policy information about sex, gender (identity/presentation), and sexual orientation and race, ethnicity and racism.

Reporting on sex and gender	*Use the terms sex (biological attribute) and gender (shaped by social and cultural circumstances) carefully in order to avoid confusing both terms. Indicate if findings apply to only one sex or gender; describe whether sex and gender were considered in study design; whether sex and/or gender was determined based on self-reporting or assigned and methods used. Provide in the source data disaggregated sex and gender data, where this information has been collected, and if consent has been obtained for sharing of individual-level data; provide overall numbers in this Reporting Summary. Please state if this information has not been collected. Report sex- and gender-based analyses where performed, justify reasons for lack of sex- and gender-based analysis.*
Reporting on race, ethnicity, or other socially relevant groupings	*Please specify the socially constructed or socially relevant categorization variable(s) used in your manuscript and explain why they were used. Please note that such variables should not be used as proxies for other socially constructed/relevant variables (for example, race or ethnicity should not be used as a proxy for socioeconomic status). Provide clear definitions of the relevant terms used, how they were provided (by the participants/respondents, the researchers, or third parties), and the method(s) used to classify people into the different categories (e.g. self-report, census or administrative data, social media data, etc.) Please provide details about how you controlled for confounding variables in your analyses.*
Population characteristics	*Describe the covariate-relevant population characteristics of the human research participants (e.g. age, genotypic information, past and current diagnosis and treatment categories). If you filled out the behavioural & social sciences study design questions and have nothing to add here, write "See above."*
Recruitment	*Describe how participants were recruited. Outline any potential self-selection bias or other biases that may be present and how these are likely to impact results.*
Ethics oversight	*Identify the organization(s) that approved the study protocol.*

Note that full information on the approval of the study protocol must also be provided in the manuscript.

Field-specific reporting

Please select the one below that is the best fit for your research. If you are not sure, read the appropriate sections before making your selection.

☒ Life sciences ☐ Behavioural & social sciences ☐ Ecological, evolutionary & environmental sciences

For a reference copy of the document with all sections, see nature.com/documents/nr-reporting-summary-flat.pdf

Life sciences study design

All studies must disclose on these points even when the disclosure is negative.

Sample size	Mouse numbers for experiments assessing UTI were predetermined based on our prior published data on UPEC titers in urine and tissue. For flow cytometry, a pilot study of mice was conducted to reduce the total number of animals used, estimate variability among animals, and evaluate procedures. In vitro experiments with 5637 cells used 3 biological replicates in each experiment as is standard in the field and was sufficient to detect statistically significant differences between treatment groups.
Data exclusions	No data were excluded from analyses.
Replication	Data from mice are from at least two independent experiments each with at lease 5 mice per group. Data from in vitro experiments with 5637 urothelial cells are from 2-3 independent experiments, each with three biological replicates per condition. All attempts at replication were successful.
Randomization	Mice were randomized into control and experimental groups.
Blinding	Investigators were blinded to group allocation during flow cytometry data collection. Blinding during bacterial infection experiments in vitro and in vivo was not possible because of the visual difference between the vehicle PBS control inoculum and the bacterial inoculum.

Reporting for specific materials, systems and methods

We require information from authors about some types of materials, experimental systems and methods used in many studies. Here, indicate whether each material, system or method listed is relevant to your study. If you are not sure if a list item applies to your research, read the appropriate section before selecting a response.

Materials & experimental systems

n/a	Involved in the study	
☐	☒	Antibodies
☐	☒	Eukaryotic cell lines
☒	☐	Palaeontology and archaeology
☐	☒	Animals and other organisms
☒	☐	Clinical data
☒	☐	Dual use research of concern
☒	☐	Plants

Methods

n/a	Involved in the study	
☒	☐	ChIP-seq
☐	☒	Flow cytometry
☒	☐	MRI-based neuroimaging

Antibodies

Antibodies used	CD45 BV510 (BD Horizon; #563891; clone 30-F11), MHCII BUV395 (BD Optibuild; #743876; clone 2G9), CD19 BUV661 (BD Horizon; #612971; clone 1D3), CD3 BUV805 (BD OptiBuild; #741982; clone 17A2), CD11b BV570 (Biolegend; #101233; clone M1/70), F4/80 BB700 (BD Optibuild; #746070; clone T45-2342), NK1/1 PE/Dazzle594 (Biolegend; #108748; clone PK136), Ly6C PE-Cy5.5 (Novus Biologicals; #N100-65413PECY55; clone ER-MP20), Ly6G AF700 (Biolegend; #127621; clone 1A8), Siglec F BV421 (Biolegend; #155509; clone S17007L), CD11c BV785 (Biolegend; 117336; clone N418)
Validation	Antibodies used were selected based on reactivity to mouse and flow cytometry application according to the manufacturer's website. For exact validation procedures and certificates, go to regdocs.bd.com or biolegend.com. Antibodies were titrated in lab to ensure optimal staining dilutions.

Eukaryotic cell lines

Policy information about cell lines and Sex and Gender in Research

Cell line source(s)	ATCC5637 cell line (aka HTB-9)
Authentication	cell line was authenticated by ATCC by STR profiling
Mycoplasma contamination	cell line received from ATCC was certified free of mycoplasma contamination
Commonly misidentified lines (See ICLAC register)	no commonly misidentified cell lines were used in this study

Animals and other research organisms

Policy information about studies involving animals; ARRIVE guidelines recommended for reporting animal research, and Sex and Gender in Research

Laboratory animals	Mus musculus C57BL/6J (#000664 Jackson Labs) wild type and 129S2-Nr4a1tm1Jmi/J (#006187 Jackson Labs), 7-8 week old
Wild animals	none
Reporting on sex	Only female mice were used because UTI are more prevalent in women and the mouse model of transurethral catheterization and UTI pathogenesis is more established in female mice .
Field-collected samples	na
Ethics oversight	All mouse experiments were approved by the Washington University School of Medicine Institutional Animal Care and Use Committee

Note that full information on the approval of the study protocol must also be provided in the manuscript.

Plants

Seed stocks	*Report on the source of all seed stocks or other plant material used. If applicable, state the seed stock centre and catalogue number. If plant specimens were collected from the field, describe the collection location, date and sampling procedures.*
Novel plant genotypes	*Describe the methods by which all novel plant genotypes were produced. This includes those generated by transgenic approaches, gene editing, chemical/radiation-based mutagenesis and hybridization. For transgenic lines, describe the transformation method, the number of independent lines analyzed and the generation upon which experiments were performed. For gene-edited lines, describe the editor used, the endogenous sequence targeted for editing, the targeting guide RNA sequence (if applicable) and how the editor was applied.*
Authentication	*Describe any authentication procedures for each seed stock used or novel genotype generated. Describe any experiments used to assess the effect of a mutation and, where applicable, how potential secondary effects (e.g. second site T-DNA insertions, mosiacism, off-target gene editing) were examined.*

Flow Cytometry

Plots

Confirm that:

☒ The axis labels state the marker and fluorochrome used (e.g. CD4-FITC).

☒ The axis scales are clearly visible. Include numbers along axes only for bottom left plot of group (a 'group' is an analysis of identical markers).

☒ All plots are contour plots with outliers or pseudocolor plots.

☒ A numerical value for number of cells or percentage (with statistics) is provided.

Methodology

Sample preparation	Bladders were aseptically harvested postmortem and digested enzymatically as described by {Atta, et al #} (Collagenase Type 1, III, VI, and Pan, DNase, Papain, and Hyaluronidase) for the 24 hpi (Fig 3B-F) experiments or described by {Mora-Bau, 2015} (Liberase, DNase) for naïve (Fig S2) and 4 wpi (Fig 3H-M) experiments. Following digestion, the samples were centrifuged and the pellets were resuspended in RBC cell lysis buffer (155 mM NH_4Cl, 10 mM $KHCO_3$) for 1 min at room temperature. The samples were then filtered through a 70 m filter, centrifuged and the pellets were resuspended in 1 mL PBS. Cell counts were obtained via hemacytometer, and 1 x 106 cells were added to FACS tubes. Cells were stained with Live/Dead Fixable Blue (Thermo Fisher Scientific; #L34962) on ice for 30 min in the dark, washed with FACS buffer (PBS containing 0.01% FBS and 0.1% sodium azide), centrifuged, and incubated with Fc Block (BD Pharmingen; #553142) for 15 min at 4 C. Antibody cocktail (see Supplemental Table 1) g was added and incubated for 45 min at 4° C covered from light. After antibody staining, the cells were washed two times in FACs buffer, filtered through 40 µM nylon mesh and analyzed on the Cytek® Aurora (Cytek Biosciences) flow cytometer. Blood was collected via cardiac puncture at the time of sacrifice and joined the bladders at the red blood cell lysis step. All flow analysis was performed using FlowJo (Treestar) software. Immune cell populations were identified according to our established gating strategy (Fig. S5).
Instrument	Cytek Aurora from Cytek Biosciences, Inc. Model number N7-00003
Software	SpectroFlo was used to collect the data and FlowJo was used to analyze the data.
Cell population abundance	Enzymatic digestion was used to carefully disassociate the bladder cells and achieve the highest yield. After doublet discrimination and selection of live cells, CD45 positive cells were gated to enrich for immune cells. The entire sample was collected to get a representative population of the whole.
Gating strategy	Doublets were eliminated by plotting FSC-A against FSC-H and gating only on the cells that fell in the positive linear area. All of the following gates were defined by using an unstained bladder sample to determine negative and positive populations.

☒ Tick this box to confirm that a figure exemplifying the gating strategy is provided in the Supplementary Information.
